# Estrogen Receptor Expression Is Associated with DNA Repair Capacity in Breast Cancer

**DOI:** 10.1371/journal.pone.0152422

**Published:** 2016-03-31

**Authors:** Jaime Matta, Luisa Morales, Carmen Ortiz, Damian Adams, Wanda Vargas, Patricia Casbas, Julie Dutil, Miguel Echenique, Erick Suárez

**Affiliations:** 1 Department of Basic Sciences, Division of Pharmacology & Toxicology, Ponce Health Sciences University-School of Medicine, Ponce Research Institute, Ponce, Puerto Rico, United States of America; 2 Department of Basic Sciences, Division of Cancer Biology, Ponce Health Sciences University-School of Medicine, Ponce Research Institute, Ponce, Puerto Rico, United States of America; 3 Public Health Program, Ponce Health Sciences University, Ponce, Puerto Rico, United States of America; 4 Department of Basic Sciences, Division of Biochemistry, Ponce Health Sciences University-School of Medicine, Ponce Research Institute, Ponce, Puerto Rico, United States of America; 5 Auxilio Mutuo Hospital, San Juan, Puerto Rico, United States of America; 6 Department of Biostatistics and Epidemiology, Graduate School of Public Health, University of Puerto Rico, Medical Sciences Campus, San Juan, Puerto Rico, United States of America; University of North Carolina School of Medicine, UNITED STATES

## Abstract

Estrogen-receptor-positive (ER+) tumors employ complex signaling that engages in crosstalk with multiple pathways through genomic and non-genomic regulation. A greater understanding of these pathways is important for developing improved biomarkers that can better determine treatment choices, risk of recurrence and cancer progression. Deficiencies in DNA repair capacity (DRC) is a hallmark of breast cancer (BC); therefore, in this work we tested whether ER signaling influences DRC. We analyzed the association between ER positivity (% receptor activation) and DRC in 270 BC patients, then further stratified our analysis by HER2 receptor status. Our results show that among HER2 negative, the likelihood of having low DRC values among ER- women is 1.92 (95% CI: 1.03, 3.57) times the likelihood of having low DRC values among ER+ women, even adjusting for different potential confounders (p<0.05); however, a contrary pattern was observed among HER2 positives women. In conclusion, there is an association between DRC levels and ER status, and this association is modified by HER2 receptor status. Adding a DNA repair capacity test to hormone receptor testing may provide new information on defective DNA repair phenotypes, which could better stratify BC patients who have ER+ tumors. ER+/HER2- tumors are heterogeneous, incompletely defined, and clinically challenging to treat; the addition of a DRC test could better characterize and classify these patients as well as help clinicians select optimal therapies, which could improve outcomes and reduce recurrences.

## Introduction

Worldwide, breast cancer (BC) is a growing health problem, both in its increasing incidence [1] and resistance to treatment [2]. Between 2008 and 2013, the worldwide incidence of BC rose by 20% [3]. Additionally, 30% of all estrogen-receptor-positive (ER+) breast tumors exhibit *de novo* resistance, and 40% of patients who initially respond to treatment will acquire resistance despite remaining ER+ [4,5].

ER status is an established therapeutic and prognostic biomarker for BC [6–10]. Although hormone receptor status is a mainstay for molecular and clinicopathological classification of BC [11–15], it provides an incomplete molecular model for classifying BC tumors. Although significant progress has been made in the clinical management of ER+ tumors, prevailing challenges are associated with their molecular heterogeneity, resistance to standard endocrine therapy, and the risk of late recurrence. These challenges are driving intense research efforts to find predictors of chemosensitivity in ER+ BC.

The majority of BC deaths ultimately occur in ER+ women. ER signaling is complex and highly regulated; it affects both proliferation and survival [16]. Understanding the scope and influence of ER signaling is challenging—including how it affects therapeutic response. Approximately 75% of all breast tumors are ER+ [17–21], but only about half of all ER+ tumors respond to anti-estrogen therapy [19,22–24]. Additionally, only 20% of those tumors stop expressing ER when they become endocrine resistant [25,26]. There is therefore an urgent need to find effective interventions [27,28].

ER signaling works through both genomic and non-genomic mechanisms that engage in crosstalk [25,29]. ER signaling has a propensity to increase proliferation, which can lead to mutations when DNA repair is dysregulated. Such dysregulation is a hallmark of breast carcinogenesis [30–34] and confers a phenotype of increased cellular division or decreased apoptosis [35]. Indeed, recent studies have documented interactions between ER and DNA damage response/repair [36,37]. Moreover, our previous work showed that DNA repair capacity (DRC) in BC patients is approximately half that of women without the disease (*p*<0.001) [38], and deficiency in the nucleotide excision repair (NER) pathway figures prominently in the pathobiology of sporadic BC [39].

The relationship between ER activation/signaling and DNA damage/repair is only starting to be investigated. This manuscript: (1) explains our recent findings regarding the association between ER status and the defective DNA repair phenotype, controlling for different variables including HER2 receptor status [38,39], (2) outlines potential mechanisms behind what we found for future mechanistic studies, and (3) proposes how our findings could form the basis for a new combination of biomarker testing that may better characterize BC tumors, which could improve diagnostic, prognostic, and therapeutic efforts.

## Results

### Study population

The average age of women with BC was 56.4 years (±12.2) at the time of initial diagnosis. All of the women were untreated BC cases. Approximately, 18.5% were younger than 45 years and 24.4% were older than 65 years. Nearly 25% of the women with BC studied reported having consumed multivitamins in the last 5 years, 17.6% reported having consumed calcium in the last 5 years. Approximately 65% were menopausal women, and 24.8% of patients had a family history of BC. About 52% (n = 127) of the women studied with BC had grade II tumors, followed by 36% of the women with grade III tumors (n = 88), only 13% (n = 31) had grade I tumors (Table 1).

**Table 1:** Characteristics of study group consisting of women with breast cancer. Women with BC history, menopausal and their tumor grade and receptor status (estrogen, HER2, progesterone) classification expressed as numerical and % values (n = 270).

Variables	Summary measures(N = 270)
	N (%)
DRC (%)*	3.05 ± 2.8
***Median DRC (P25*, *P75)***	2.1 (1.24%, 4.07%)
**DRC (tertiles)**	
**< 1.6%**	90 (33.3)
**1.6–3.2%**	90 (33.3)
**> 3.2%**	90 (33.3)
**Age**	
**< 45**	50 (18.5)
**46–55**	79 (29.3)
**56–65**	75 (27.8)
**> 65**	66 (24.4)
***Median Age (SD)***	56.4 ± 12.2
**History of breast cancer**	
**Yes**	67 (24.8)
**No**	203 (75.2)
**Menopausal status**	
**Yes**	174 (64.4)
**No**	96 (35.6)
**Multivitamin consumption in the last 5 years**	
**Yes**	66 (25.2)
**No**	204 (74.8)
**Calcium consumption in the last 5 years**	
**Yes**	46 (17.6)
**No**	224 (82.4)
**Tumor grade**	
**I**	31 (12.6)
**II**	127 (51.6)
**III**	88 (35.8)
***Missing***	24
**Receptor status**	
**Estrogen Receptor**	
**Negative**	73 (27.0)
**Positive**	197 (73.0)
**HER2 Receptor**	
**Negative**	206 (76.3)
**Positive**	64 (23.7)
**Progesterone Receptor**	
**Negative**	100 (37.0)
**Positive**	170 (63.0)

* Value of % DNA repair capacity (DRC) measured in lymphocytes expressed as mean ($\bar{\text{X }}$), ± 1 standard deviation (SD) and median values including percentile 25 (P25) and percentile 75 (P75).

**Fig 1:**
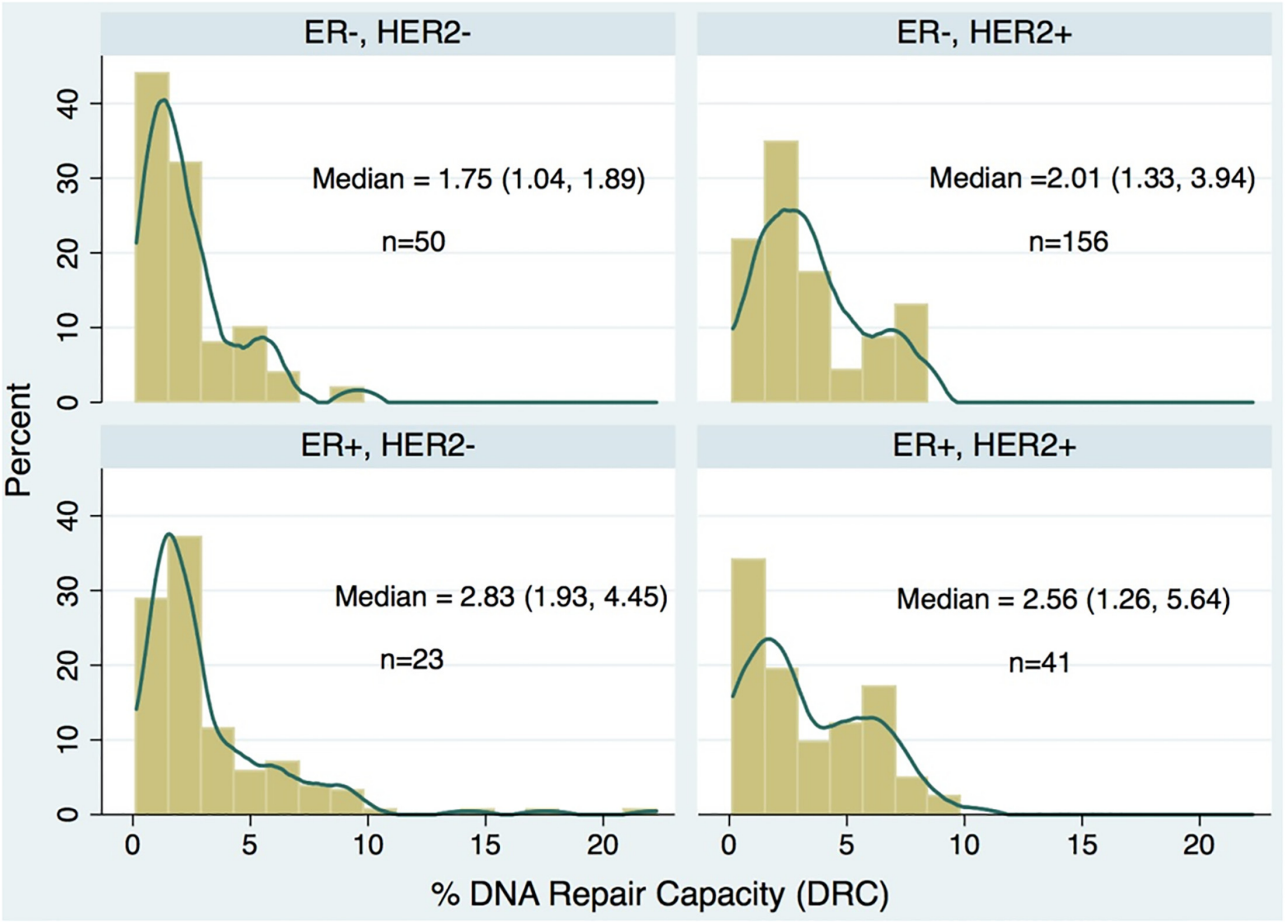
Distribution of levels of % DNA repair capacity (DRC) in 270 women with breast cancer separated in four groups according to estrogen receptor (ER) and HER2 status. The height of the rectangle denotes the percentage of women with a specific %DRC level. The line denotes the Kernel density estimation (KDE) to show the smoothing distribution of the %DRC. Between parentheses indicates percentile 25 and percentile 75.

The overall mean value DRC was 3.0% (±2.8% S.D.) and the median DRC value was 2.1%, with an interquartile range of 2.8% (75th percentile minus 25th percentile) (Table 1). Nearly 73% of the BC patients had tumors classified as ER positive, 23.7% had HER2 positive tumors and 63% were progesterone positive (Table 1). When the DRC distribution was described by HER2 (+,-) and ER (+,-) status among women with BC, different patterns were observed. In all four combinations of the categories of these two receptors, the DRC values showed a positive skew (were highly concentrated at low values) (Fig 1). The highest median DRC value (2.83%) was in the ER-HER2+ group, the ER-HER2- group had a DRC value (1.75%), representing a reduction of approximately 38%. However, among HER2 negatives, a higher number of women had low DRC values, independently of the ER status (Fig 1).

### Ordinal Logistic regression model

The ordinal logistic model was utilized to analyze the data with different cutoff points in the DRC according to the HER2 status. This was done in order to more precisely assess the relationship between DRC expressed as a categorical variable in tertiles and ER status. Different patterns were observed, as presented in Table 2. These were divided into two groups:

**Table 2:** Magnitude of the association between tertiles of levels % DNA repair capacity (DRC) according to estrogen receptor (ER) and HER2 receptor status (n = 270).

HER2	ER		%DRC		Adjusted^#^	Adjusted^#^	Adjusted^#^
		<1.6%	1.6–3.2%	>3.2%	${\text{OR}}_{\text{ER}-\text{vs}.\text{ER}+}^{\left(\leq 1.6\right)}$	${\text{OR}}_{\text{ER}-\text{vs}.\text{ER}+}^{\left(\leq 3.2\right)}$	${\text{OR}}_{\text{ER}-\text{vs}.\text{ER}+}^{\left({\text{low DRC level}}^{\left(1\right)}\right)}$
Negative	Negative	24	14	12	2.23* (1.13, 4.38)	1.42 (.67, 2.99)	1.92* (1.03, 3.57)
(n = 206)	Positive	46	60	50	1	1	1
Positive	Negative	5	8	10	0.38 (0.11, 1.32)	0.92 (0.31,2.73)	0.67 (0.25,1.79)
(n = 64)	Positive	15	8	18	1	1	1
Total	Negative	29	22	22	1.48 (0.84, 2.61)	1.26 (0.70, 2.27)	1.38 (0.83, 2.29)
(n = 270)	Positive	61	68	68	1	1	1

^#^Adjusted for menopausal status, breast cancer history age, multivitamin and/or calcium consumption in the last 5 years

*P-value < 0.05

(1) Only 1 OR is reported because the proportional odds assumption was met (p>0.05).

### Women with HER2 negative breast cancer tumors

The results indicate that the likelihood of having a DRC below 1.6% among women with ER- tumors is 2.23 (95% CI: 1.13, 4.68) times the likelihood of having a DRC below 1.6% among ER+ women. After adjusting for different potential confounders, this excess likelihood was statistically significant (p<0.05). When the cutoff point of the DRC of 3.2% is used (Table 2), the likelihood of having a DRC below 3.2% among ER- women is 1.42 (95% CI: 0.67, 2.99) times the likelihood of having a DRC below 3.2% among ER+ women, even adjusting for different potential confounders; however, this excess was not statistically significant (*p*>0.05). When we assessed the proportional odds assumption of the ordinal logistic model, the results were not significant (*p*> 0.05); hence, only one OR can be estimated without depending on the cutoff point. Therefore, the results indicate that the likelihood of having low DRC values among ER- women is 1.92 (95% CI: 1.03, 3.57) times the likelihood of having low DRC values among ER+ women, even adjusting for different potential confounders; this excess likelihood was statistically significant (*p*<0.05).

### Women with HER2 positive breast cancer tumors

Women with HER2 BC tumors showed the opposite pattern, the likelihood of having low DRC values among ER- women is 33% (OR: 0.67, 95% CI: 0.25, 1.79) lower than the likelihood of having low DRC values among ER+ women, even adjusting for different potential confounders; however, this reduction was not statistically significant (*p*>0.05).

## Discussion

We found that DRC and ER levels are associated and that this association is modified by HER2 receptor status in women whose BC tumors are HER2-. This association suggests that DRC and ER levels are linked: as DRC levels increase, ER levels also increase, and vice versa. Although previous studies have established that a defective DNA repair phenotype is commonly found in women with BC [38–40], to our knowledge, this is the first study to show that ER status is associated with a defective DNA repair phenotype. Our previous study (Matta et al. 2012) using a case control design with 824 women showed the usefulness of DRC level as a measure of BC risk. For every percent unit of decrease in DRC, there is 64% more likelihood of having BC. Thus, the subtle differences in DRC in women with BC divided into the four groups presented in Fig 1, provide additional evidence on how DRC is associated with BC risk. For example, the two groups with the lowest DRC (ER-/HER2-, ER+/HER2-) represented 76% of the study group comprised of 270 women. DNA repair genes, particularly those involved in base excision repair may have a significant effect on estrogen-driven breast cancer specific survival (BCSS) as recently shown by Abdel-Fatah et al. (2014). They studied 1,406 women with ER positive early stage BCs with 20 years long term clinical follow-up data. Multivariate Cox proportional hazards model was used to calculate a DNA repair prognostic index and correlated to clinicopathological variables and survival outcomes. Key base excision repair proteins including XRCC1, APE1, SMUG1 and FEN1 were independently associated with poor BCSS [36].

### Background related to findings

Most ER+ breast tumors are classified as luminal [13–15,41]. Luminal A is characterized by ER+/PR+/HER2-/low Ki-67 [42] and typically responds well to endocrine therapy [28]. In contrast, Luminal B is either HER2+ or HER2- but is ER+/PR+/high Ki-67 [42]; such tumors are complex diagnostically and therapeutically [28]. Luminal A tumors are considered as more differentiated, indolent, and sensitive to endocrine therapy, whereas Luminal B tumors are more aggressive and resistant to endocrine therapy [43]. Indeed, Lips’ 2012 analysis of biopsies from 211 treatment-naïve ER+/HER2- primary breast tumors could not define a subgroup that would be most likely to benefit from neoadjuvant therapy, much less predict chemosensitivity or treatment outcome. Biopsies of the same patients taken post-treatment but prior to surgery were equally unrevealing [41].

Despite today’s availability of comprehensive gene signature assays (see review by Issa et al. 2014) and our increasing knowledge of which genes are dysregulated in BC and how, the information has not always resulted in increased clinical utility or better prognostic tools [44]. Santarpia’s 2013 research on a 145-gene expression profile to try to characterize ER+/HER2- subgroups yielded interesting but inconclusive information [37]. But problems exist even with genes that have been studied in depth for years. For example, *EGFR* is amplified in many BCs, and its crosstalk with ER signaling is well established. Yet large randomized trials of treatment-naïve ER+/HER- patients did not respond any better to treatment when an EGFR signaling inhibitor was coupled with an aromatase inhibitor [41]. Similar results occurred in studies using a fibroblast growth factor receptor inhibitor [41]. ASCO’s 2014 guidelines acknowledge this knowledge gap and note that no optimal first- or second-line treatment exists for advanced ER+/HER2- BC [8]. Collectively this underscores the need for better molecular classification [19,23,41,45], and ER+/HER2- breast tumors may be the “canary in the coal mine” pointing us to a more effective predictive model: a combination of receptor assays and DRC. Even though research has made great strides in the molecular characterization of DNA repair pathways—and efforts to map NER and two other pathways were awarded 2015 Nobel Prizes in Chemistry—our knowledge still remains incomplete. Because of this we propose that the combination of molecular signatures data, including the DNA repair prognostic index recently developed by Abdel-Fatah et al. (2014), with DNA repair phenotypic data can enable more accurate diagnostic and therapeutic decisions [36].

### Biological plausibility of our findings

In healthy people, ER signaling generally downregulates DNA damage response (DDR) [46] while simultaneously promoting proliferation [46,47]. Seventy percent of ER activity is governed by highly regulated genetics that keep ER’s influence in check [23]. However, dysregulated ER signaling confers a phenotype of increased proliferation or decreased apoptosis [35]. *Sustained* ER signaling permits accumulation of low-level DNA damage, an event documented early in tumorigenesis [48].

ER activation leads to transcriptional responses both in genes with and without estrogen response elements. Additionally, ER activation can elicit non-transcriptional cellular responses, all of which favor cell proliferation and survival [21,49]. This diversity points to traditional (canonical) genomic signaling in the nucleus, as well as plasma-membrane-bound (non-canonical) signaling. The former involves ER binding directly to a cognate ligand on the promoter region of a gene. In the latter, ER “tethers” to “pioneer factors” that enable ER to influence pathways that eventually act on downstream target transcription factors (such as ER’s interactions with *VEGF* and *AP-1*) [21]. Both types of activities result in altered gene expression, and both can lead to point mutations and uncontrolled cellular proliferation—despite the former being genomic while the latter is not [45]. More importantly, sustained ER signaling can lead to greater expression of proliferation-related genes that may rely less on expression of traditional ER genes but more on alternate pathways for activation and tumor growth [28].

Our finding that women with BC and ER negative tumors have lower DRC values among HER2- seems counterintuitive at first. However, this molecular response may be an early event in tumorigenesis—perhaps ER’s attempt to induce DNA repair to offset increasing replication errors of early mutagenesis.

This raises the question of what factor(s) could modulate that association, and how might that affect treatment options? If such factors could be determined, more effective biomarker panels and therapeutic regimens could be devised. Although the DRC assay is currently used primarily as a method of estimating BC risk [38], its combination with hormone receptor status as described here may expand its applications.

Despite the variety of gene signature assays available today, a 2014 analysis [50] shows that even the most comprehensive assay is not cost-effective in pinpointing optimal adjuvant treatment or outcomes for ER+ BCs [50]. New combinations of molecular markers such as DRC plus hormone receptor status may allow better tumor differentiation within the same molecular subtype—and lead to more effective treatment.

### Why only ER+/HER2- tumors?

Breast cells with estrogen-induced DNA damage can either delay damage response/repair or can promote cell survival at the expense of repair [51]. So why this DRC/ER relationship would be detectable only in the ER+/HER2- subpopulation is a tantalizing question.

ER appears to influence virtually every DNA repair pathway [46,48,52–54]. While direct evidence for some of those pathways is sparse, dysfunction and downregulation of the NER pathway is prominent and well-documented in BCs [38–40]. Abnormal expression of two NER proteins (RPA, a damage sensor; and PCNA, a progressivity factor) is associated with ER+/HER2- breast tumors [37].

Two linchpins for ER’s influence on DNA repair pathways may be p53 and PI3K. ER appears to have a bidirectional, yin-yang relationship with p53, which influences many repair pathways. But more evidence for how ER can shift repair signaling appears to be associated with the PI3K pathway—a superhighway for survival signaling [26,55,56]. Hyperactivation of the PI3K-Akt-mTOR pathway is common in BC [57]. Dysregulated expression of growth factor receptors including ER can activate the Ras/Raf/MEK/ERK pathway, Ras/PI3K/PTEN/Akt/mTOR and other signaling pathways, creating a self-sustaining feed-forward loop of persistent signaling [58], which could lead to resistance to hormonal therapy [59]. S1 Fig depicts our vision of how estrogen affects DNA repair.

### Strengths and limitations of this study

To our knowledge, we are the first to explore the relationship between DRC and ER in this manner. Additionally, we believe we are the first to explore how HER2 status affects the DRC/ER relationship. This novel approach has great potential utility for personalized medicine. For example, with ER emerging as a contributor to dysregulated DNA repair; it makes sense to include testing for a DNA repair phenotype along with hormone receptor status testing. Based on our results, our proposal using these two types of testing in combination can provide further molecular characterization of breast tumors—with possible insights into better treatment choices—particularly in the more clinically challenging BCs including, but not limited to ER+/HER2- cases. Our study has robust sample size and power [38]; results were consistent after adjustment of different sources of variability (menopausal status, history of BC, and HER2 status) in the ordinal logistic regression model.

## Conclusions

The heterogeneity of ER+ tumors, their unequal response to anti-estrogen therapy, and ER’s capacity to influence gene expression collectively point to the need for more comprehensive molecular characterization of BC tumors. In BC, adding a test for DRC to a genome signature allows identification of the defective DNA repair phenotype and may enhance our capacity to predict which Luminal B tumors are likely to be more aggressive. This allows selection of better treatment regimens, especially for diagnostically and therapeutically complex molecular BC subtypes such as ER+/HER2-. Our findings indicate a previously undetected, subtle difference in ER+ tumors, which could also help explain the lack of correlation between end points among some clinical trials for these tumors. The availability of single-cell and single-molecule assays puts the exciting possibility of better molecular profiling within our grasp.

## Methods

### Patient Recruitment

The study was approved by the Ponce Health Sciences University Institutional Review Board (IRB #120207-JM). Patients for this study were selected from our larger BC study (1,181 patients and controls; recruited 2006–2012), which has been previously described [38,60,61]. The cases selected for this study were women with primary diagnosis of BC and were recruited from gynecological and oncology clinics throughout Puerto Rico.

Inclusion criteria were patients who: (1) were recently diagnosed histopathologically with primary BC, (2) were treatment-naïve (had not received chemotherapy, blood transfusions, or radiotherapy), and (3) had pathology reports that included hormone receptor information. We obtained consent forms that allowed us to interview the participants, obtain blood samples, and review their pathology reports. Among the cases, 344 women were eligible to participate in the study; however, only 270 had complete hormone receptor status information. Therefore, our final study group comprised 270 BC cases.

### DNA Repair Capacity (DRC) Measurements

The host cell reactivation (HCR) assay with a luciferase reporter gene that we utilized to measure DRC levels in lymphocytes has been described in previously published molecular epidemiological studies of cancer [40,62–68]. We have previously published details about the variability, stability of plasmid transfection and differences between cryopreserved versus fresh blood samples for the HCR [38]. This assay measures the total DRC of transfected lymphocytes. Results reflect the host cells’ overall repair capacity, although HCR primarily detects activity of the nucleotide excision repair (NER) pathway [38].

### Blood collection and isolation of lymphocytes from women

We used peripheral blood samples to obtain lymphocytes, which were separated, purified and grown from each patient sample [38]. Those cells were used as surrogate markers of the patients’ overall DRC [69,70]. Approximately 30 mL of peripheral blood was obtained from each participant and stored in heparinized tubes. The lymphocytes were then isolated by the Ficoll gradient technique and suspended in 2 mL of freezing media containing 10% dimethyl sulfoxide, 40% RPMI 1640 medium, 50% fetal bovine serum, and 1% antibiotic/antimycotic. Aliquots were stored in a −80°C freezer for 1–3 weeks. The lymphocytes were later thawed in batches of 5–7 samples for the HCR assay (details follow). Collection periods were approximately the same for patients and women without BC because recruitment was conducted concurrently.

The HCR was performed on the peripheral blood lymphocytes to measure *in vivo* DRC, as described in previous studies [38,40,60,61,71]. The late Dr. Lawrence Grossman (Johns Hopkins School of Public Health, Baltimore, MD) provided the luciferase plasmid for the HCR assay and the protocol for its use. A nonreplicating plasmid expression vector (pCMVluc) of 4,863 base pairs was genetically engineered to contain a bacterial luciferase reporter gene that is not present in a mammalian cell. The gene was damaged by ultraviolet C radiation (254 nm) exposure in a controlled, quantitative manner (dose—response curve) so that the level of its expression was a direct measure of the repair capacity of the host mammalian cell. The plasmid construct containing the luciferase gene (LUC) was irradiated at 0, 350, and 700 J/m2 using a 254-nm UVC lamp (38). This plasmid construct and its validation have been described previously [72]. The controlled, quantitative UV exposure produced a dose—response curve so that the level of its expression was a direct measure of the repair capacity of the host mammalian cell. After transfection into lymphocytes, repair-transcription-blocking damage was introduced exogenously on foreign DNA; then overall DRC was measured via HCR [71]. This approach measured the unaffected phenotype, which reflects the cells’ inherent DRC, measured primarily in terms of their NER activity [71]. Keeping the time constant for the lymphocytes to complete the repair mirrored the true cellular process [70].

To calculate the DRC, undamaged plasmid DNA was compared to repair of *in-vitro*-damaged plasmid DNA; the results were expressed as the percentage of residual luciferase reporter gene expression (% luciferase activity in luminescence units). The amount of gene expression reflected DRC, expressed as a percentage. A detailed description of the assay, including separation of DRC by tertiles, is found in Matta *et al*. 2012 [38]. This study builds upon our laboratory’s 17 years of experience in performing the HCR assay to measure DRC and its validation of the sensitivity, specificity, and usefulness as a measure of BC risk [38].

### Hormone Receptor Status

We reviewed the patients’ medical records to collect receptor status data on estrogen (ER), progesterone (PR) and human epidermal growth factor receptor 2 (HER2). Ten private laboratories in Puerto Rico performed the receptor status assays on patients’ formalin fixed tumor biopsies, using immunohistochemistry (IHC) methods per ASCO (American Society of Clinical Oncology) and CAP (College of American Pathologists) guidelines [10,73]. ER and PR analyses included the percentage of positive-staining cells, the intensity of staining (weak, moderate, or strong), and an interpretation (“receptor positive” meant ≥1% of invasive tumor cells stained positive for ER/PR; “receptor negative” meant <1% of invasive tumor cells stained positive for ER/PR) [10].

HER2 testing also utilized IHC-based assays. All laboratories used FDA-approved IHC assays. When an assay yielded equivocal results (2+ or 1+), Fluorescence In Situ Hybridization (FISH) was used to determine whether those samples were HER2+ or HER2-, based on the quantity of HER2 gene copies per nucleus [10]. For our analysis, we categorized HER2 status as a dichotomous variable of ‘‘positive” (all 3+ results) or ‘‘negative” (2+ to 0 results).

### Statistical Analysis

We used descriptive statistics to summarize quantitative variables; while for categorical variables we used percentages in each category of these variables. To assess the possibility of selection bias, a significance test was performed using the normal approach to compare the mean DRC among women with complete (n = 270) versus incomplete (n = 74) receptor status information. The results from this comparison did not show any significant difference (*p*>0.05).

An ordinal logistic regression model (OLRM) was used to assess the relationship between DRC and ER status [74]. The OLRM is an extension of the logistic regression model that applies to dichotomous dependent variables, allowing for more than two (ordered) response categories. In order to apply this model to our analysis, the DRC was categorized in different groups, using as a cut-off points the observed DRC tertiles (<1.6%, 1.6–3.2%, >3.2%). The expression of this model is as follows:

$$log\frac{P_{\leq k}}{P_{>k}}=\beta_{0k}-\beta_{Ek}*ER+\sum \beta_{i}C_{i}$$

where P_≤k_ indicates the prevalence of women with DRC equal or below tertile k, P_>k_ indicates the prevalence of women with DRC above tertile k, β_Ek_ indicates the coefficient associated to ER when the *k*th-DRC tertile is used, ER is a dummy variables to indicate the ER status (+, -), and β_i_ indicates the coefficient associated to each potential confounding variables, C_i_. This model provides an estimate of the magnitude of the association ($OR_{ER-vs.ER+}^{\left(\leq k\right)}$) between DRC and ER at different cut-off point of the DRC, as follows:

$$OR_{ER-vs.ER+}^{\left(\leq k\right)}=e^{{\hat{\beta }}_{EK}\pm 1.96SE\left({\hat{\beta }}_{EK}\right)}$$

where $SE\left({\hat{\beta }}_{EK}\right)$ indicates the standard error of ${\hat{\beta }}_{EK}$. However, if the assumption of proportional odds ($OR_{ER-vs.ER+}^{\left(\leq 1.6\right)}$ = $OR_{ER-vs.ER+}^{\left(\leq 3.2\right)}$) is met, then only one OR is estimated without depending of the cutoff point of the DRC. Our data showed that this assumption was met (*p*>0.1); therefore, only one estimation of the OR was reported.

## Supporting Information

S1 Fig Potential pleotropic effects of estrogen on proliferation, DNA damage and DNA repair capacity in breast cancer cells. Estrogen can have a variety of effect on the tumor cells some of which may involve DNA repair signaling specially among HER2(-) tumors. (A) In the canonical pathway, ER binds to the promoter regions of genes involved in cell cycle progression. (B) Non-canonical pathways involve many other signaling cascades such as p53, mTOR and ERK which can also lead to increased proliferation. Both of these pathways can lead to low levels of DNA damage that may “switch on” DNA repair mechanisms (evaluated in in this study in the patients’ circulating lymphocytes).
(DOCX)

## Abbreviations

BC: breast cancer
ER: estrogen receptor
HER2: HER2neu receptor
DRC: DNA repair capacity
NER: nucleotide excision repair
OR: odds ratio
OLRM: Ordinal logistic regression model
