# Supplementary material for: Estrogen Receptor Expression Is Associated with DNA Repair Capacity in Breast Cancer
Source: PLoS One. 2016 Mar 31;11(3):e0152422. doi: 10.1371/journal.pone.0152422 (PMC4816515; doi:10.1371/journal.pone.0152422)
Supplement: S1 Fig — Estrogen can have a variety of effect on the tumor cells some of which may involve DNA repair signaling specially among HER2(-) tumors. (A) In the canonical pathway, ER binds to the promoter regions of genes involved in cell cycle progression. (B) Non-canonical pathways involve many other signaling cascades such as p53, mTOR and ERK which can also lead to increased proliferation. Both of these pathways can lead to low levels of DNA damage that may “switch on” DNA repair mechanisms (evaluated in in this study in the patients’ circulating lymphocytes). (DOCX) [file pone.0152422.s001.docx]

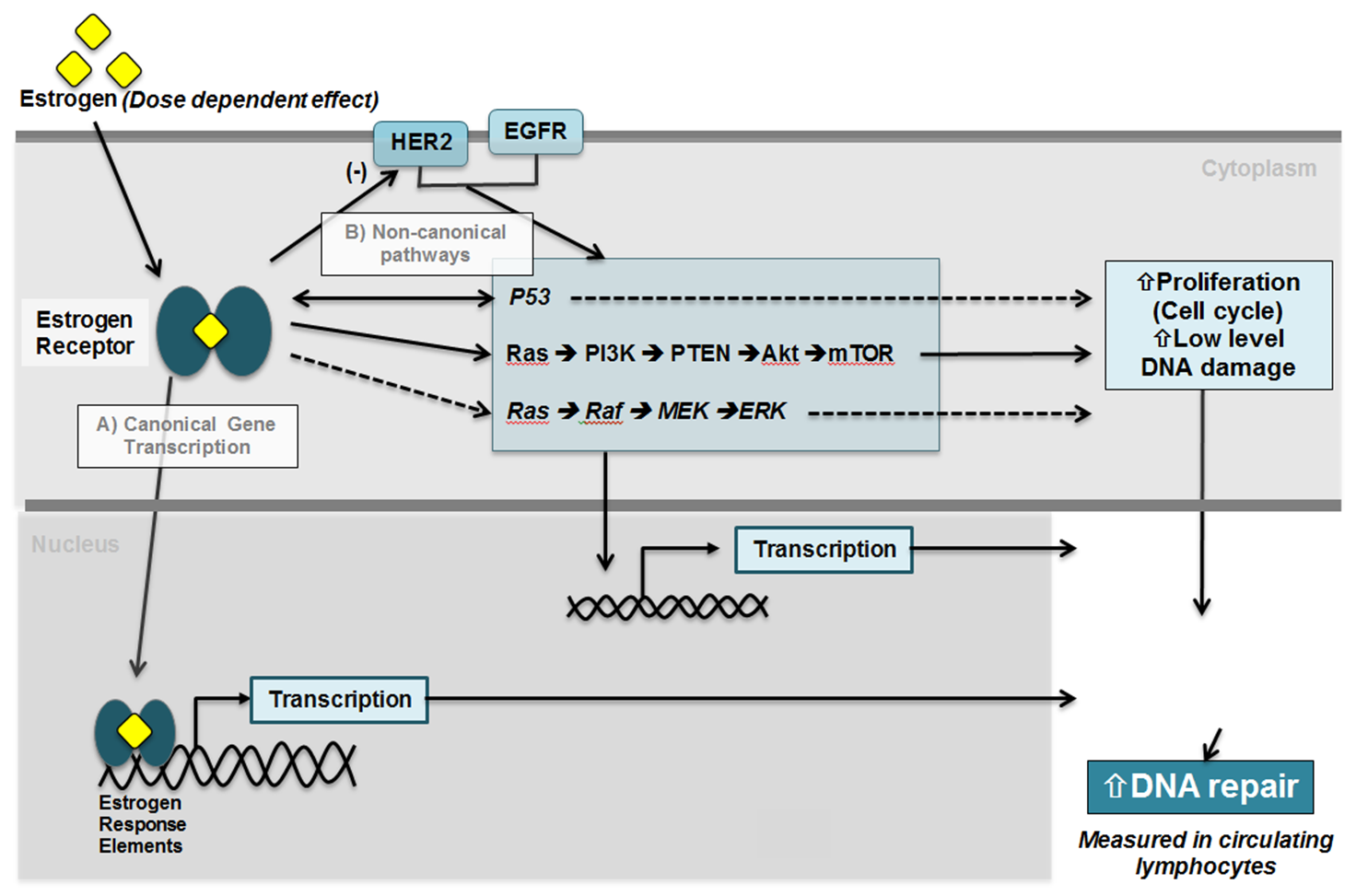


**S1 Fig. Potential pleotropic effects of estrogen on proliferation, DNA damage and DNA repair capacity in breast cancer cells.**

Estrogen can have a variety of effect on the tumor cells some of which may involve DNA repair signaling specially among HER2(-) tumors. (A) In the canonical pathway, ER binds to the promoter regions of genes involved in cell cycle progression. (B) Non-canonical pathways involve many other signaling cascades such as p53, mTOR and ERK which can also lead to increased proliferation. Both of these pathways can lead to low levels of DNA damage that may “switch on” DNA repair mechanisms (evaluated in in this study in the patients’ circulating lymphocytes).
